# The Foes of Hospital Economy

**Published:** 1920-11-27

**Authors:** Sydney Dunstan

**Affiliations:** The Royal Victoria Infirmary, Newcastle-on-Tyne


### THE EDITOR'S LETTER-BOX.

**Correspondence on all subjects is invited, but we cannot in any way be responsible for the opinions expressed by our correspondents, who must give their name and address as a guarantee of good faith, but not necessarily for publication. Correspondents are reminded that brevity of style and conciseness of statement greatly facilitate early insertion.**

### "The Foes of Hospital Economy."

*To the Editor of* The Hospital.

Sir,—With your kind permission I should like to reply briefly to Mr. Hocking's letter in a recent issue.

Mr. Hocking states that he published a paper similar to mine eleven or twelve years ago. I was not aware of this, and perhaps Mr. Hocking will be good enough to state when and where it appeared.

I do not claim that there is anything original in the suggestion of co-operation and standardisation, but I do contend that thousands of pounds can be saved annually by making better use of the laboratory equipment usually found in all our big hospitals. In support of this statement I may mention that in this hospital, working on the lines already suggested, the expenditure was curtailed by no less than £655 for the year 1919, and this year it will be considerably more, which has been done without the engagement of any additional labour or extra expenditure on new machinery. This work is especially referred to by the House Committee in the annual report of the Royal Victoria Infirmary for the year 1919, page 30. Mr. Hocking states that pharmacists of his acquaintance have quite enough to do in discharging their legitimate duties. I challenge this statement, for I claim that the pharmacist's time, experience, and training can be utilised to much greater advantage to the hospital than as at the present time, and his reference to a miniature Crosse and Blackwell factory is all nonsense and requires no further comment.

I see nothing new in Mr. Hocking's suggestions for dispensary reforms. These, I believe, are well known, and have been in existence for some considerable number of years. In his concluding remarks, he suggests that a committee should be set up to inquire into and report upon the subject of co-operative buying. If Mr. Hocking had taken advantage of the committee, which was set up in 1913 on the lines he now suggests, perhaps he would be more enlightened on the subject than he appears to be at the present time, and I cannot see that any useful purpose would be served by such a committee being set up now. Moreover, if its members were of the same views as Mr. Hocking, the policy pursued would not be one of co-operation or reconstruction.

In conclusion, I still claim that co-operative buying and standardisation are practical and sound. What finer example can we have of this than the great establishment controlled by Sir Jesse Boot and his staff? I had the opportunity some time ago of visiting their headquarters in Nottingham, and one could not but admire the great organisation they have, for the supplying of all their branches throughout the country.—Yours faithfully,

The Royal Victoria Infirmary, Sydney Dunstan.
Newcastle-on-Tyne.
November 16, 1920.

### "The Future of the Hospitals."

*To the Editor of* The Hospital.

